# Analysis of Trauma Intensity during Surgical Bone Procedures Using NF-κB Expression Levels as a Stress Sensor: An Experimental Study in a Wistar Rat Model

**DOI:** 10.3390/ma11122532

**Published:** 2018-12-12

**Authors:** Marcos Barbosa Salles, Sergio Allegrini Jr., Marcelo Yoshimoto, Leticia Pérez-Díaz, José Luis Calvo-Guirado, Sergio Alexandre Gehrke

**Affiliations:** 1 Department of Anatomy, Universidade de São Paulo, Cidade Universitária, São Paulo, SP 05508-900, Brazil; marcsall@gmail.com (M.B.S.); sergiojr@usp.br (S.A.J.); marcelo.yoshimoto@gmail.com (M.Y.); 2 Laboratorio de Interacciones Moleculares, Facultad de Ciencias, Universidad de la Republica, Iguá 4225, Montevideo, Canelones 11400, Uruguay; letperez@gmail.com; 3 Department of Research in Oral Implantology, Universidad Católica San Antonio (UCAM), 30107 Murcia, Spain; jlcalvo@ucam.edu; 4 Department of Biotechnology, Biotecnos—Technology and Science, Cuareim 1483, Montevideo, Canelones 11100, Uruguay

**Keywords:** NF-κB, surgical bone trauma, dental implants, vitaminic compound, irrigation drilling

## Abstract

**Aim and objectives**: It is well known that the transcription factor NF-κB regulates multiple aspects of innate and adaptive immune functions and functions as a pivotal mediator of inflammatory responses. In the present study, we evaluated the trauma generated (inflammatory reaction) after osteotomy bone surgical procedures and placement of implants in the femoral cortical bone of Wistar rats. Surgical stress was evaluated measuring the release and activation of the NF-κB factor. **Materials and Methods**: Rats were divided into four groups (n = 10) and submitted to different surgical treatments: Control Group (G1 group), only bone perforation was performed without irrigation; Implant Group (G2 group), a titanium implant was inserted after bone perforation without irrigation; Irrigated Group (G3 group) perforations were performed with intense irrigation; and Vitaminic Compound Group (G4 group) surgical perforation was performed without irrigation and a vitaminic compound containing the principal ions present in the natural bone structure was used to fill the bone defect. All animals were euthanized six hours after the surgical procedure and NF-κB levels were determined through immunohistochemical stain followed by direct counting of labeled and unlabeled osteocytes. **Results**: Among different treated groups, the overall mean of the NF-κB positive cell count in all positions were higher for G1 group (33.4 ± 2.45 cells). NF-κB values were lower in the G2 group (28.9 ± 2.70 cells), whereas in the G3 group (24.3 ± 2.72 cells) as well as in G4 group still lesser NF-κB positive cells were counted (26.5 ± 2.60 cells). **Conclusions**: The results here presented suggest that maneuvers performed during osteotomy procedures can significantly affect inflammation levels. The NF-κB activation during the surgical procedures can be minimized and/or controlled thought the adequate irrigation or application of adequate substances.

## 1. Introduction

During surgical procedures for the fixation of endosteal screws or implants, the drilling management may cause stress by the friction generated by surgical drills in the bone, hampering the proper healing of the tissue [1,2,3]. Local heat generated in the drilling location is considered the main cause of surgical trauma (stress), and thus, to minimize the consequences during this procedure, intensive irrigation methods to control the temperature are required [3,4]. Moreover, some studies showed that drill geometry (conical or cylindrical) seems to be an important factor not only in heat generation during implant site preparation [5,6], but also in the healing response of bone tissue [1]. It has also been reported that oscillating fluid flow reduces stress in osteoblasts modulating bone turnover [7].

After surgical bone procedures (i.e., drilling and/or implant installation), intracellular chemical signals are released into the cell in response to stress, leading to a dynamic regulation of signal transduction into the cell nucleus [8,9] to trigger the expression of specific genes for monitoring a suitable cellular response to external variations in order to develop specific and adaptive responses of cells or tissues to external stimulus [10,11,12,13]. Haddad and colleagues [13,14] have suggested the activation of two proteins as the main stress sensors: 1-α HIF-1α (hypoxia-inducible factor-1α) and NF-κB (nuclear factor kappa-beta). The first is a transcription factor expressed under hypoxia [15,16], and the second is a widely studied dimeric, redox-sensitive, transcription factor involved in the regulation of a large number of genes controlling various aspects of the immune and inflammatory response [17] that has been associated with several cellular functions, including stress-induced responses and survival [10,16,18,19]. This transcription factor also participates in bone remodeling processes by inducing increased osteoclast formation and activity also participating in certain aspects in osteoblast and chondroblast activities [20]. Moreover, NF-κB has also been considered by many authors [21,22,23,24] as an important marker of cellular and tissue damage and it has also been proposed to be a sensor for oxidative stress [25].

It is well established that stress induced by reactive oxygen species (ROS), which increase with an inflammatory state, can affect bone homeostasis by stimulating osteoclast differentiation and bone resorption [26,27]. However, the therapeutic use of antioxidants after bone damage is still very controversial, and their mechanism of action when applied in clinical studies (laboratory animals or in humans) is still unknown. Moreover, antioxidant supplementation in vivo could cause hepatic damage and/or renal toxicity [28,29]. To avoid this toxicity, different researchers associate the antioxidant to a scaffold, allowing its release in a controlled manner, and thus, controlling the deleterious action of ROS, improving tissue repair [30,31,32]. The use of these biomaterials in animals with induced nerve trauma resulted in a complete tissue regeneration [33,34]. In this context, a biomaterial composed of different vitamins and minerals associated to a hydrogel, here referred to as vitamin compound, was also evaluated after surgical trauma.

In the present work, we analyzed the effect of different bone surgery procedures in the stress produced by the bone tissue management during orthopedic surgeries in the cortical bone of Wistar rats. Trauma intensity was evaluated through quantification of NF-κB levels used as a stress sensor. In this context, the NF-κB activation peak was analyzed after different procedures including intense saline irrigation, the insertion of a dental implant after bone damage, and the application of a vitaminic compound in surgically induced traumatic lesions. Results here presented suggest that surgical bone trauma can be controlled during operative procedures such as intense irrigation or the insertion of an implant and/or the application of substances that control the release of inflammatory mediators.

## 2. Materials and Methods

### 2.1. Materials

The vitamin compound was prepared using the following components: β-carotene (BASF, Ludwigshafen am Rhein, Germany), α-tocoferol (BASF), complex B (F. Hoffmann-La Roche AG, Basel, Switzerland), calciferol (BASF), selenium salts (BASF), zinc salts (Merck KGaA, Darmstadt, Germany), magnesium salts (Merck KGaA), calcium salts (Merck KGaA), phosphoro salts (Merck KGaA), glutamic acid (BASF), soy lecithin (BASF), hydrolyzed collagen (BASF), glycosaminoglycan sulfate (BASF), and chondroitin sulfate (BASF). The selenium, zinc, and magnesium salts varied between 10 and 15% of the total weight of the active portion, and selenium in a proportion of 20% to 30% of the relative weight of the other magnesium and zinc salts, these with proportions of 1.8% and 1.2%, respectively. β-carotene and α-tocopherol vitamins ranged from 15 to 20%, with α-tocopherol having a ratio of 64% to β-carotene. Complex B had a proportion ranging from 20 to 30% of the total weight of the active portion of the material, with vitamins B1 and B6 maintaining a proportion of 30% of the complex and glutamic acid 50% of vitamin B6. Calcium and phosphorus salts were used in the proportion of 30% of the total weight of the active portion and in the proportion of 54% between them. Vitamin D (calciferol) was added at the end of the process, at a ratio of at most 40 parts per million of the total weight of the material. Hydrolyzed collagen (gelatin), glycosaminoglycan sulfate and chondroitin sulfate were added in the ratio of 60, 20, and 20%, respectively.

Commercially pure titanium implant (ASTM-F67) were manufactured by Implacil De Bortoli (São Paulo, Brazil), with dimensions of 1.8 mm in diameter and 6 mm in length.

Both materials (vitaminic compound and implants) were previously prepared, packed, and sterilized with gamma radiation, with an intensity of 25 KGy at room temperature (Embrarad, São Paul, Brazil).

### 2.2. Animal Model and Care

Forty Wistar male rats weighing approximately 350 g were used in this experimental study. The study was approved by the animal experiment Ethics Committee (#107/03) of the Department of Anatomy of Biological Science Institute of the University of São Paulo (São Paulo, Brazil). The experiment was performed in accordance with the Brazilian guidelines and regulations, i.e., followed the standards of animal welfare in accordance with the Sociedade Brasileira de Ciência de Animal de Laboratório, SBCal (http://www.cobea.org.br) and the Brazilian federal law regulating issues related to animal research [35]. The animals were randomized to receive different treatments (www.randomiztion.com). Four groups were created (*n* = 10 animals per group) with the following treatments: Control Group (G1 group) in which bone perforations were made without irrigation; Implant Group (G2 group) where perforations were made without irrigation and an implant was inserted in the bone lesion; Irrigated Group (G3 group) where the perforations were performed with abundant irrigation using sterile physiological solution (sodium chloride 0.9%); and Vitaminic Compound Group (G4 group) where perforations were performed without irrigation and the generated defect was filled by the organic compound.

The animals were kept in a controlled temperature room at 21 °C following the international lighting standard (periods of 12 h of darkness and 12 h under artificial light) using lighting timers.

### 2.3. Animals Surgery

The surgical procedure consisted of the perforation of the proximal femur. During the procedure, the animals were kept under deep anesthesia for 60 to 90 min. Besides, each animal received sedation and muscle relaxant through the administration of intramuscular injection (2-2-xylidine)-5,6-dyhidro-4H-1,3-thyazyn chlorate (Rompum, Bayer, São Paulo, Brazil) (5.0 mg/kg), acepromazine (Acepran 1%-Univet, São Paulo, Brazil) (0.75 mg/kg). For general anesthesia, intramuscular injection at 35 mg/kg of ketamine (Ketamina, Agener, Union Chemical National Framacêutica SA, São Paulo, Brazil) was administered. Initially, the trichotomy was performed followed by an antisepsis with iodopolvidone solution. The incision was made in the skin and posteriorly in the fascia, in the proximal–distal direction, followed by drilling with 1.8 mm diameter drills. The perforations were performed without irrigation (G1, G2, and G4 groups) to increase the surgical trauma and consequently the inflammatory reaction. For the G3 group, the perforations were performed using intense irrigation with sterile saline solution. After perforation, muscle tissue and skin were closed through nylon 4.0 sutures. Euthanasia was performed after 6 h of the surgical procedure since this point in time corresponds to the peak time of NF-κB activation [36].

### 2.4. Sample Preparation

After euthanasia, the samples were immersed in 10% buffered formalin fixative solution (buffer phosphate 0.1 M pH 7.4). All samples were fixed at 4 °C for 7 days under established conditions [31]. Briefly, samples were washed in running water for 12 h at room temperature and serial dehydration was performed with ethanol solutions starting from 70%, 80%, 90%, 95%, and 100% for 72 h each wash. Dehydration was carried out at −20 °C. Samples were then incubated twice with xylol at −20 °C, for a period of 24 h to remove fat and facilitating the penetration and imbibition of the resin Technovit resin 7200 VLC (Kulzer & Co, Wehrhein, Germany), being finally polymerized [37].

Samples were then cut in a microtome (Exakt cutting equipment, Exakt Apparatebeau, Norderstedt, Germany) and then worn and polished using a metallographic machine (Panambra, São Paulo, Brazil) [38]. The final thickness of the samples varied between 10 and 15 μm.

To enable the immunohistochemical stain the acrylic was removed from the samples using the solvent 2-Methoxyethyl acetate (Sigma–Aldrich Inc, Darmstadt, Germany) before being rehydrated.

### 2.5. Immunohistochemical Process

**Figure 1:**
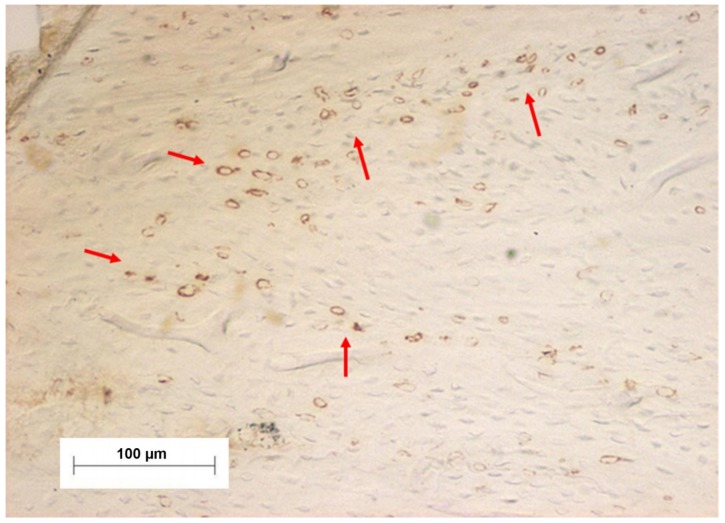
Image showing red arrows where there were positive immunohistochemical reactions (NF-κB labelling) of cells (brown color).

Samples were fixed on glass slides and treated with 20% 3-aminopropyltriethoxy silane (Sigma Chemical, St. Louis, MI, USA). For antigen retrieval the slides were immersed in citric acid solution pH 6.0 for 30 min at temperatures between 95 °C and 98 °C. Slides were incubated with polyclonal anti-NF-κB antibody diluted 1/75 (Zymed Laboratories Inc., San Francisco, CA, USA) containing BSA 1%, washed and incubated in secondary antibody conjugated to peroxidase (Dako Corporation, Carpinteria, CA, USA). The immunohistochemical reaction was developed using DAB (3,3′ diaminobenzidine, Sigma Chemical, St. Louis, MI, USA) 0.025% as a chromogen, which is oxidized by peroxide in the presence of hydrogen peroxide resulting in a deposition of a brown color, as showed in the sample image in Figure 1 where labelled cells (brown color) were determined as the positive NF-κB expression. After 3 min of color development, slides were dehydrated and mounted with Permount (Fisher Scientific, Fair Lawn, NJ, USA).

### 2.6. Data Collect

**Figure 2:**
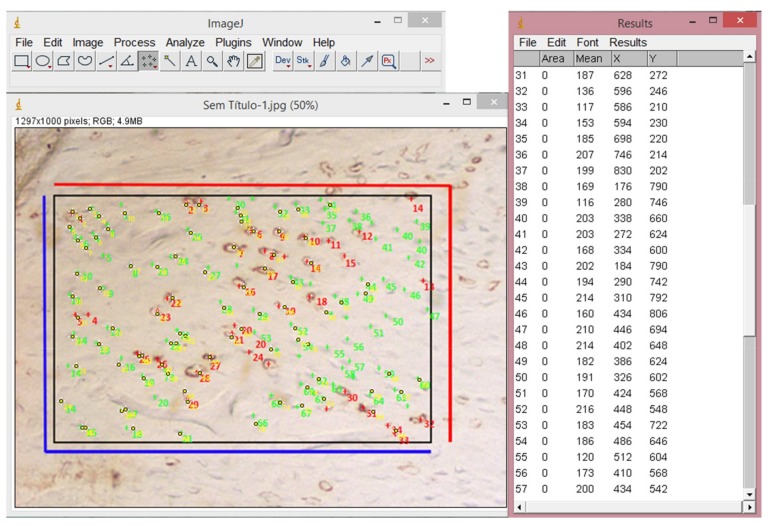
Image of a previously standardized area was included in the captured image to quantify the labeled (in red) and unlabeled (in green) cells using the software ImageJ.

Images were obtained through optical microscopy (Nikon, Eclipse E-1000, Tokyo, Japan) with a magnification of 10× and then analyzed using ImageJ (National Institutes of Health, Bethesda, Maryland, USA) Version 1.50i for Windows (Figure 2).

**Figure 3:**
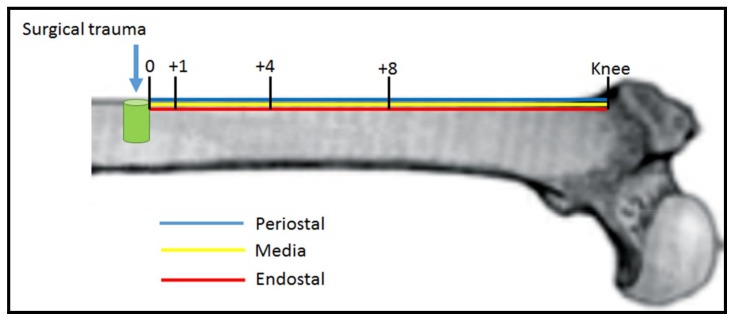
Schematic figure showing position and regions used for cell counting. Distances in mm from the performed bone lesion (Zero, +1, +4, 8 and knee) are shown. Each position was assayed in three different regions: periostal (blue), media (yellow), and endostal (red).

Labeled and unlabeled cells were counted 3 times by 2 different researchers (Marcos B. Salles and Sergio A. Gehrke). Since no significative differences were found among the three positions tested for NF-κB levels (Periostal, Media, and Endostal), data was presented as the mean number of these three positions. NF-κB quantification was then analyzed at different positions from the site of perforation: location of the perforation (p0), 1 mm from perforation (p1), 4 mm from perforation (p4), 8 mm from perforation (p8) and knee (pk). Positions and regions of interest used for cell analysis in each sample are indicated in the scheme in Figure 3.

Bone microdamage generated by the compression of the implants during their installation (bone microcrack) was analyzed in G2 samples by optic microscopy.

### 2.7. Statistical Analysis

The sample size was based on a power of 85% to obtain a *p*-value of 0.05. For a desired power of 85%, with a difference between means of 25 points, and a standard deviation of 8 points, the sample size minimum in each group resulted in 4 animals. Data were expressed as means ± SD. Comparison of groups (G1, G2, G3, G4) as well as positions (p0, p1, p4, p8, pk) were performed with the ANOVA repeated measures (using the two-factor study with a repeated measure on one factor under Greenhouse–Geisser correction) through a dedicated software (MedCalc, Ostend, Belgium). The Mann–Whitney test was used as a second step to identify which positions were significantly different in the same group. Significance was accepted at a level of *p* < 0.05.

## 3. Results

The operative surgical sites healed without incident. In performed postoperative controls, no healing problems were observed, presenting adequate evolution in the period of evaluation. All animals were available for histological analysis.

The average and standard deviation of NF-κB levels in each group determined in each position from the bone lesion are summarized in the Table 1. The Greenhouse–Geisser correction was 0.74 indicating homogeneity of variances. The test between-subject effects (groups) revealed a significant statistical difference (F = 48.97; *p* < 0.001). Besides, the test of within-subject effects (positions) also revealed a significant statistical difference (F = 23.73; *p* < 0.001). Finally, the interaction between group x position also revealed a significant statistical difference (F = 15.44; *p* < 0.001).

Samples from the G1 group, where the perforations were made without irrigation, showed in average the highest NF-κB positive cell count (33.4 ± 2.45 cells). The lowest NF-κB positive cell count was observed in groups treated either with saline irrigation (24.3 ± 2.72 cells) or when vitaminic compound was applied (26.5 ± 2.60 cells) after bone lesion (G3 and G4 groups, respectively) suggesting a significative reduction of the trauma. However, when the implant was inserted in the lesion (G2 group) the decrease in the mean number of NF-κB positive cells (28.9 ± 2.70 cells) was intermediate between control G1 group and G3/G4 group.

**Figure 4:**
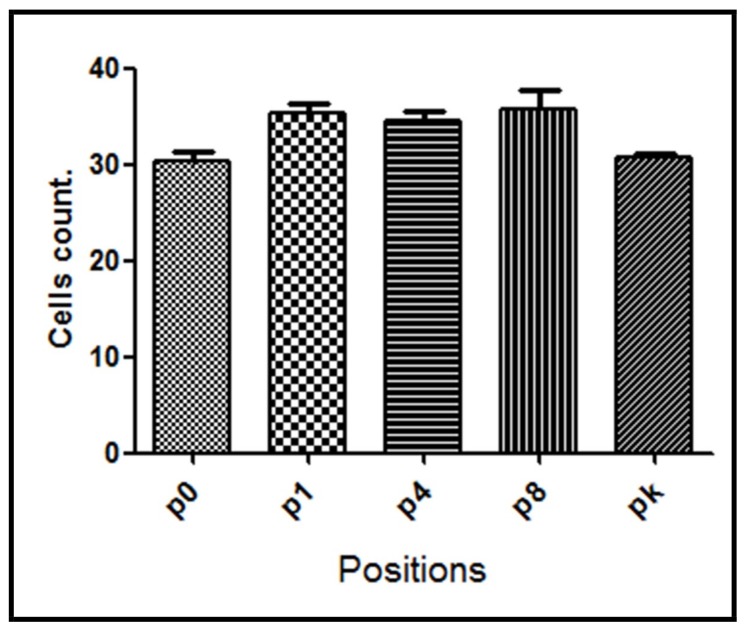
NF-κB positive cell count for G1 group evaluated at different positions from the bone lesion. The mean number with the corresponding standard deviation is shown for each point.

NF-κB expression was also assessed between positions in the same group. For G1 group, significant differences were found (*p* = 0.0032) between assayed positions being NF-κB expression augmented in positions p1, p4, and p8 compared with position p0 and pk (knee) (Figure 4).

**Figure 5:**
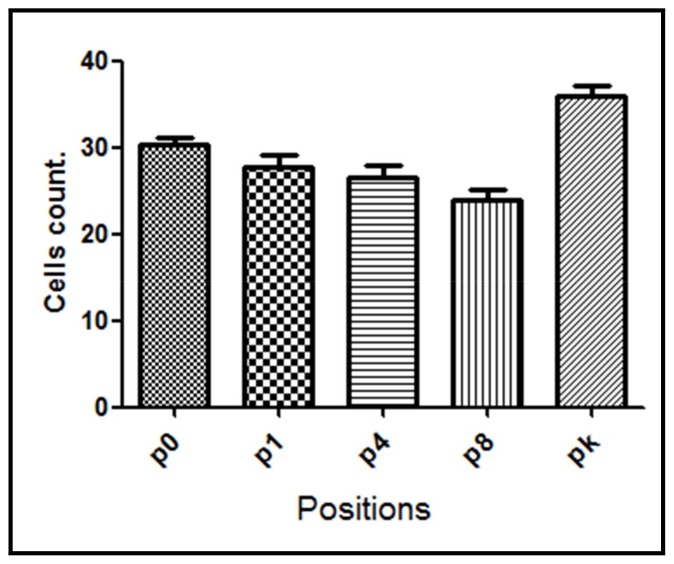
NF-κB positive cell count for G2 group evaluated at different positions from the bone lesion. The mean number with the corresponding standard deviation is shown for each point.

For the G2 group, higher NF-κB values were observed in the perforation site (position p0) while lower NF-κB values were observed as we move away from the drilling site until reaching the knee position where NF-κB values increased. This finding is in agreement with a higher induction of trauma at the perforation site due to both the drilling without irrigation and also the implant placement (Figure 5). This trauma decreases while moving away from the perforation site up to the knee, where it increases again. Overall, a significant statistical difference was observed between assayed positions (*p* < 0.0001).

**Figure 6:**
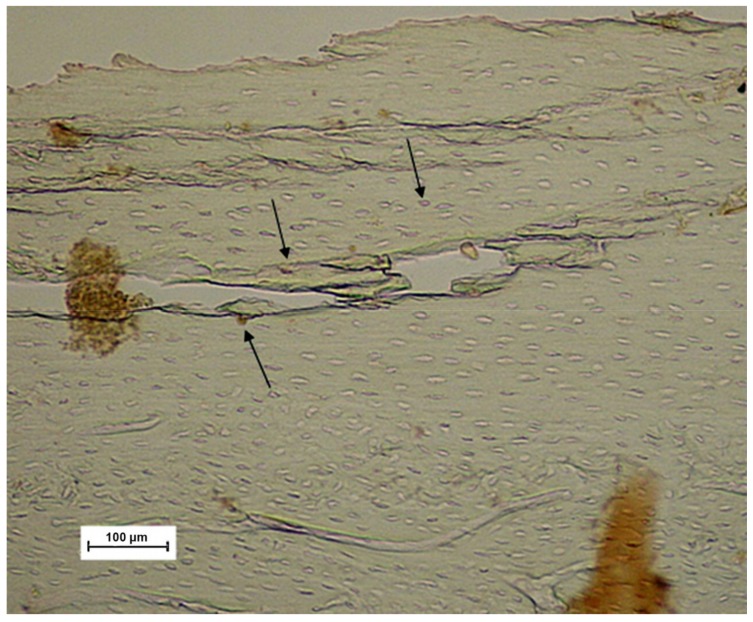
Representative image of the sample from the G2 group showing a microfracture in the bone. Black arrows denote the immunolabelled cells.

Moreover, the insertion of an implant in the cortical bone resulted in a microcrack caused by compression of the implant during its insertion into the perforation of the bone tissue (Figure 6).

**Figure 7:**
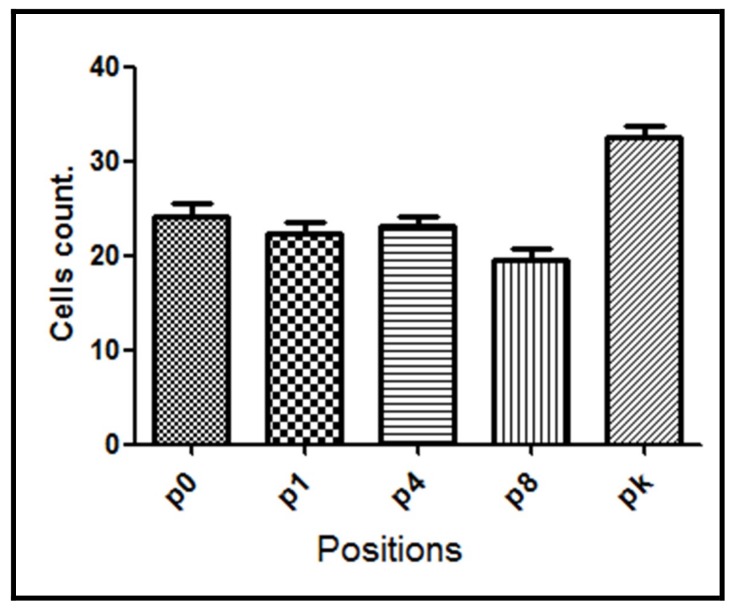
NF-κB positive cell count for the G3 group evaluated at different positions from the bone lesion. The mean number with the corresponding standard deviation is shown for each point.

Regarding samples from the G3 group, also a significant difference was observed between assayed positions (*p* < 0.0001) with a reduction in the NF-κB immunoreactivity observed in the 4 positions assayed from the bone lesion (p0, p1, p4, and p8) with respect to the knee position (Figure 7), where NF-κB positive cell count reached similar levels to the G1 and G2 groups.

**Figure 8:**
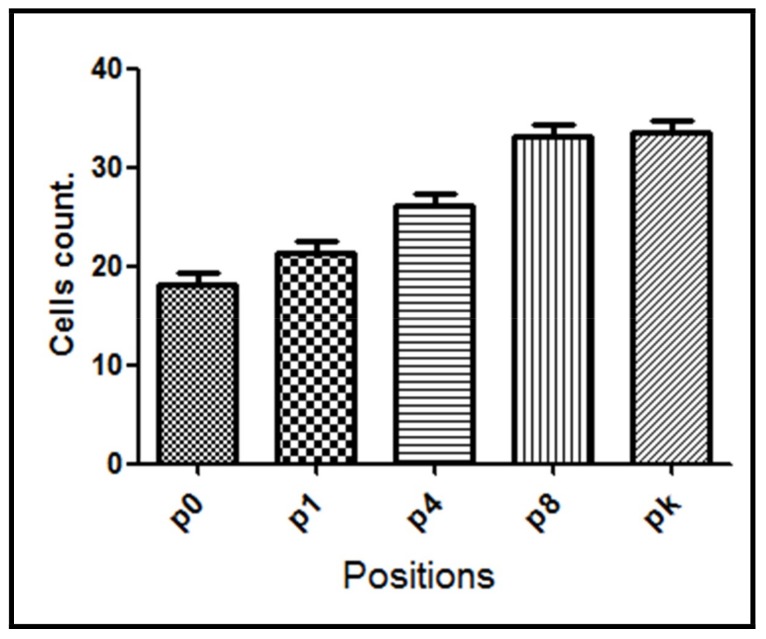
NF-κB positive cell count for the G4 group evaluated at different positions from the bone lesion. The mean number with the corresponding standard deviation is shown for each point.

Finally, with respect to the G4 group, a significant statistical difference was also observed between assayed positions (*p* < 0.0001). Our results show a significant reduction in the NF-κB immunoreactivity observed mainly in the perforation site (position p0) when compared with the other three groups (Figure 8). The NF-κB levels increased from this position until reaching NF-κB positive cell counts similar to those observed in the G1 group at p8 and pk positions. These findings suggest a gradual increase of trauma from the perforation site.

## 4. Discussion

Overall, on the basis of the hypothesis that transcription factor NF-κB could be considered as a sensor of cellular and tissue stress, in the present study, NF-κB was selected as a stress marker and the presence of this protein as well as its peak of activation were evaluated through a specific technique developed by our group in different bone trauma situations [36,37]. Increased immunoreactivity for NF-κB observed in Wistar rat osteocytes after bone damage supports the idea of its significant role in bone remodeling and bone repair, regulating physiological processes involved in osteoclastic reabsorption and osteoblastic formation.

In all studied animal groups, where different procedures were performed after bone damage, NF-κB activation was induced at the damage position and propagated along the cortical area, suggesting that bone tissue cells allow the transference of NF-κB from adjacent cells after stress induction. This mechanism could be related directly with osteocyte ultrastructure with its complex network communicating between adjacent cells and with osteoblasts located in the periosteum membrane. This is in agreement with the existence of a biophysic ligation between osteocytes and osteoblasts promoted by gap junctions previously reported [37,39]. The propagation of NF-κB activation from the proximal region of Wistar rat femurs after a surgical lesion can also be due to the presence of some other signaling diffusible molecules that can travel along adjacent cells [40], but also, this mechanism can be related to the oscillation of a fluid flow [7].

Bone microcracks were observed in the implant group G2, where a titanium implant was inserted after bone lesion in the lesion position. These bone fractures have already been observed and reported before [41,42,43]. Measured NF-κB levels at lesion position were similar to the levels in the control G1 group in spite of the high extra mechanical stress induced. However, NF-κB levels decrease progressively as we move away from the site of the injury until reaching the knee where the levels increase significantly. Perhaps, the introduction of implants immediately after the bone damage, could led to a stabilization of the oscillation of fluid flow inside the channels, with the consequent reduction of NF-κB delivery. The same finding was observed for the G3 group (irrigated group), where a slight decrease of the immunoreactivity for NF-κB was also detected as we move along the injury region from position zero decreasing progressively until the distal position at position p8 where an increase in NF-κB levels at the knee region was observed suggesting a reduction of the stress caused by high saline irrigation.

The slight reduction of NF-κB along cortical bone in G2 and G3 groups can be also explained by the oscillating fluid [7]. This observation reinforces the involvement of fluid flow in the propagation of NF-κB activation throughout the cortical bone as well as the importance of the cortical bone as a manager of the bone remodeling. Moreover, communicating junctions facilitate also the passage and propagation of the ions Ca++ through osteocytes allowing intracellular communication [44], and thus, inducing stress responses [45]. When an implant is inserted, Ca++ passage is impaired and ion propagation through channels is reduced. Maybe this can explain the reduction in immunoreactivity for NF-κB in this group.

The increase in NF-κB levels at knee position in G2 and G3 groups, far from surgical lesion, is in discrepancy with the increase of NF-κB under stress conditions, where the higher levels of NF-κB would be expected at the region of bone lesion (position p0) and not as far as in the knee region. This finding can be explained by the existence of a threshold of trauma response that must be overcome to trigger and propagate the generalized response from the knee region. This hypothesis is based on the basis that the shear flow caused by trauma is a mechanical issue, so any displacement of the flux would be able to activate NF-κB at distant regions, as well as other distant proteins so the response would not be restricted to a region or position.

For animals in the G4 group, the lowest levels of NF-κB were observed at the lesion position, itself reinforcing the inhibitory effect of antioxidants in NF-κB activation. This inhibition is reduced at distant positions from the injury, with NF-κB levels being progressively higher, suggesting a stress reduction mainly at the injury site and its surroundings.

Through the assessment NF-κB levels as a stress sensor, we observed a significant decrease in damage when irrigation was applied or when the vitaminic compound was added in the lesion after surgical injury. Considering that the surgical trauma induces the increase of inflammatory reaction and its consequences on the cicatricial response of the tissues, our results contribute to the development of therapeutic strategies based on NF-κB inhibition for the treatment of the inflammatory diseases [46]. It would be interesting to analyze the effect of both treatments together in the reduction of inflammation and stress after bone damage.

## 5. Conclusions

Through the analysis of the differences between three bone treatments after surgical procedures we found that:

1. The installation of an implant at the bone perforation site did not result in an increase of inflammatory response, since no increase in NF-κB levels were evidenced at the perforation site compared to a control group.
2. The irrigation process in bone perforation procedures resulted in a decrease of NF-κB levels, evidencing a reduction in trauma and inflammation processes.
3. The vitaminic compound constitutes a promising alternative in the control of the surgical trauma, since a decrease in NF-κB levels were also observed.

## Figures and Tables

**Table 1:** Average and standard deviation (±SD) of cell count with positive NF-κB expression of each group in each position (p). Distances in mm from the performed bone lesion (p0 = local of osteotomy, p1 = +1 mm, p1 = +4 mm, p8 = +8 mm, and pk = knee).

Group	p0	p1	p4	p8	pk	Average
**G1**	30.4 ± 2.24	35.4 ± 2.38	34.5 ± 2.56	35.8 ± 4.07	30.7 ± 0.98	33.4 ± 2.45
**G2**	30.3 ± 2.03	27.4 ± 2.97	26.6 ± 3.00	24.0 ± 2.68	35.9 ± 2.85	28.9 ± 2.70
**G3**	24.0 ± 3.47	22.4 ± 2.67	23.1 ± 2.29	19.5 ± 2.47	32.6 ± 2.70	24.3 ± 2.72
**G4**	18.2 ± 2.36	21.2 ± 2.75	26.2 ± 2.48	33.2 ± 2.45	33.5 ± 2.94	26.5 ± 2.60

p0 = position 0; p1 = position 1; p4 = position 4; p8 = position 8; and, pk = knee position.
